# 87 The Impact of Albumin Administration in Pediatric Burn Resuscitation

**DOI:** 10.1093/jbcr/iraf019.087

**Published:** 2025-04-01

**Authors:** Chinaemelum Akpunonu, Katherine Bergus, Brenna Rachwal, Kelli N Patterson, Renata Fabia, Rajan Thakkar, Dana Schwartz

**Affiliations:** Burn Center at Nationwide Children’s Hospital; Ohio State University, Wexner Medical Center; Nationwide Children’s Hospital; Ohio State University, Wexner Medical Center; Nationwide Children’s Hospital; Nationwide Children’s Hospital; Nationwide Children’s Hospital

## Abstract

**Introduction:**

Pediatric patients are at greater risk of developing shock during initial burn resuscitation than adult patients due to higher body-surface-area for size. Judicious use of fluids during resuscitation is crucial to decrease mortality and morbidity. To decrease excessive crystalloid volume during burn resuscitation, our center created a guideline in 2015 for patients deemed difficult to resuscitate, which replaces 1/3 of the lactated ringer hourly infusion rate with 5% albumin.

**Methods:**

We retrospectively reviewed patients admitted to our American Burn Association-verified pediatric burn center between 2008-2024 with ≥15% Total Body Surface Area (TBSA) burn who were deemed “difficult to resuscitate”, defined as requiring increase in hourly crystalloid infusion rate >40% of initial rate in response to urine output < 0.5-1.5 mL/Kg/hour. Patients were excluded if they died during admission, or were not treated according to our guideline, either because they received albumin prior to 2015 or did not receive albumin after 2015. Comparison of outcomes of the pre- and post-2015 protocol groups were made using Fisher’s exact and Wilcoxon rank sum test. A p-value < 0.05 was considered significant.

**Results:**

We identified 170 burn patients admitted to the intensive care unit, of which 31 patients met difficult to resuscitate criteria for analysis. Seventy percent of these patients were male. Burn mechanisms included flame (65%) and scald (35%). The pre-2015 group included 8 patients with a median age of 4.50 years (IQR 2.80-10.00) and the post-2015 group had 23 patients with a median age of 5.00 years (IQR 3.00-9.00) p = 0.840. The ratio of length-of-hospital-stay to TBSA burn was shorter among those who received albumin (1.20 [IQR:0.77-1.57] vs 1.29 [IQR:1.29-2.41] days p= 0.027). In addition, patients who received albumin received less total volume of intravenous fluid within the first 48 hours (12.42 [IQR 8.5713.76] vs. 7.45 [IQR 6.32-9.15] mL/kg/TBSA p= 0.033) and had lower lactate at 48 hours [1.70 [IQR 1.65-2.15] vs. 0.95 [IQR 0.73-1.25] mmol/L p= 0.021). While not reaching statistical significance, median days on a ventilator were lower in the post-protocol albumin group ((17.50 [IQR 3.00-21.5] vs 5 [0.00-9.00] p= 0.380).

**Conclusions:**

The addition of albumin to pediatric burn resuscitation reduced total IV volume infused, was associated with shorter hospital length of stay, and may have affected ventilator days. Multi-institutional studies are needed to validate these findings using a larger sample size and across different critical care settings.

**Applicability of Research to Practice:**

A resuscitation pathway that incorporates albumin for pediatric burn patients who are challenging to resuscitate decreases the risk of over resuscitation with crystalloid. Further research on early administration of albumin in this population may lead to mitigation of adverse clinical outcomes.

**Funding for the Study:**

N/A

